# Imagine a healthy lifestyle for all: Early years nutrition and physical activity to prevent obesity

**DOI:** 10.1038/s41430-022-01230-2

**Published:** 2022-11-04

**Authors:** Andrew P. Hills

**Affiliations:** School of Health Sciences, College of Health and Medicine, University of Tasmania, Launceston, Tasmania Australia

**Keywords:** Obesity, Obesity

## Introduction

Wouldn’t it be nice, with the wisdom of hindsight, to draw on some of the lifestyle experiences of our past to enable a healthier and more productive future for all young people? Rather than engineering (consciously or unconsciously) physical activity out of the lifestyle of youngsters and perpetuating an inactive phenotype and its attendant health consequences, we should assert the benefits of nutrition and physical activity for the growing child more aggressively. Imagining a simpler lifestyle may well be one of the key platforms to underpin a healthier future. Profound improvements in the *status quo* could be realised by greater investment in the health and wellbeing of young children and maximising the plasticity of the developing brain to foster human capital. Many historical examples underscore the power of early and concerted investment in young people, including the Heckman curve developed by Nobel laureate economist, James J. Heckman. Heckman postulates a significant return on investment can be realised for society by supporting early childhood learning (https://heckmanequation.org/).

Throughout my adult life I’ve been fascinated by the potential social and economic advantages of investing in human capital through the health and education of our young, and my inspiration to provide all children with quality early life experiences has stemmed from many sources. Imagine what the world would look like if the growth and development of all children, irrespective of background, was optimised through quality nutrition and physical activity across the formative years?

My crystal ball posits that we have much to learn from the past, including the notion that we would benefit from a return to the simpler aspects of life, things experienced in less technologically ‘advanced’ times in our history. How might we draw on earlier wisdom to help solve the multitude of humankind’s current health dilemmas, including obesity in childhood? This includes the suggestion that a better understanding of the processes of growth and maturation would greatly assist in managing obesity in the early years. [1]

I’ve been working in the obesity field for so long I may legitimately refer to my career as an abject failure. In essence, I commenced working in obesity prevention and management long before it was ‘fashionable’ only to find that decades later, the global prevalence of paediatric overweight and obesity continues to rise. Interesting parallels may be drawn between the scale of the obesity pandemic, declining global levels of physical activity, and corresponding increases in sedentary behaviours. We urgently need to harness our collective wisdom for greater impact, including through more progressive collaborative initiatives such as the Healthy Living for Pandemic Event Protection (HL - PIVOT) Network facilitated by close colleague, Ross Arena (https://ahs.uic.edu/physical-therapy/healthspan/hl-pivot-healthy-living-for-pandemic-event-protection/).

## Early life, education and the Pacific North-West

My passion for optimising the physical growth and development of children stems from my own early experiences. An enthusiastic athlete but average high-school scholar, I was constantly drawn to sport with my number one love being Australian Rules football. As a young person and school Prefect, I had no idea what area of study I should pursue, so in my final year of high-school, I decided to ‘try out’ for a place in a new 4-year concurrent Bachelor of Education degree being offered at the University of Tasmania. As a competent athlete with no family role model in education but strong support from loving parents, I was one of only 20 recipients of a prestigious scholarship to university and kickstart a career as a Health and Physical Education teacher. First in family to attend university, I relished the opportunity to combine my love of sport and physical activity with a burgeoning interest for working with others. Early in my undergraduate program, I knew I was destined to further my studies and leave the island, often referred to as ‘the edge of the world’, and pursue another goal, overseas travel.

My early career was dotted with great opportunities, and I vividly recall peers intimating that I must have been born with the proverbial ‘spoon in my mouth’. The truth is that I never shied away from hard work and despite my less than stellar academic credentials, was dogged in my determination to do my family proud. I attended a wonderful state (public) school on the Derwent River south of Hobart, Taroona High, completed a teaching practicum there and was subsequently rewarded with my first teaching appointment at the same school. However, my sojourn as a high-school teacher was short-lived and within a few years I commenced my long academic career at the entry level of Senior Tutor.

In 1980, I was awarded a Rotary Foundation Graduate Fellowship to undertake a Master of Science at the University of Oregon (UO) in Eugene (like Claude Bouchard and many other luminaries). In fact, a raft of prominent Australian academics completed higher degree research at the UO in the 1970s and 80s. This group is sometimes affectionately referred to as the Oregon ‘mafia’ and includes John Bloomfield, the first full professor of Human Movement Science at an Australian university, and Tony Parker, the prime mover in the progression of Exercise and Sport Science in the country. The original home of Nike and the famous Athletics West running club, Eugene was frequented by many top athletes of the day, including now infamous distance runners, Alberto Salazar and Mary Decker. Hayward Field at the UO is also the traditional home of the US Olympic Track and Field trials and was the site of the 2022 World Athletic Championships. During my time at Oregon, I had the luxury of training twice daily – when I could still run!

My primary interest in *auxology* (the science of physical growth and development), was the launching pad to my career focus on physical activity and health and honed my passion for obesity research. I was inspired by a host of influential scholars and some of their books on physical growth and related fields were prized possessions in my personal library. These included *Growth at Adolescence* (JM Tanner, 1962); [2] *Growth and Development of Children* (GH Lowrey, 1978); [3] *Physical Activity: Human Growth and Development* (GL Rarick 1973) [4] and later, *Growth, Maturation and Physical Activity* (Robert M Malina & Claude Bouchard, 1991) [5] and the Second Edition with Oded Bar-Or (2004). [6]

At the UO, one of the doyens of physical growth and development, Jan Broekhoff had a significant influence, not least of which because of his role in early longitudinal growth studies, including the Medford Boy’s Growth Study developed by H. Harrison Clarke. Broekhoff hailed from The Netherlands along with those involved in the Amsterdam Longitudinal Study, including Willem van Mechelen. The power of capturing multiple snapshots of individuals across the growing years underscored the early significance of such foundational pieces of work and was particularly illuminating, along with the fine characters responsible. These included Albrecht Claessens and the late Gaston Beunen, both involved in the Leuven Growth Study.

I returned to Australia in 1982 but soon after, left my home state again to commence a PhD in Anatomical Sciences at the University of Queensland (UQ) in Brisbane.

## The Brisbane years

My postgraduate journey was long and arduous as I completed my PhD (1990) part-time while I worked as a lecturer. Supervised by mentor Tony Parker, my PhD thesis focused on the *Locomotor Characteristics of Pre-pubertal Children with Obesity* and helped to frame my career-long interest in life course aspects of cardiometabolic and musculoskeletal health.

The 1980s in Australia saw the beginning of the demise of school Health and Physical Education and removal from its rightful place as a centrepiece of the curriculum. Of note, this coincided with the development of the new Human Movement Studies discipline. In many universities, departments morphed over time into what is now more commonly referred to as Exercise and Sport Science. During this period, the Australian Association for Exercise and Sport Science (AAESS) was formed and later, having splintered from Sports Medicine Australia (SMA), Exercise and Sport Science Australia (ESSA) became the premier professional body.

Sadly, the changing face of the profession resulted in some collateral damage, including the unintentional impact on physical activity and nutrition for health and wellbeing. Who would have predicted that in 2022, thousands of Exercise Scientists and Clinical Exercise Physiologists would be accredited in Australia? Similarly, who could have foreseen the dramatic reductions in quality school Health and Physical Education offerings? How did we allow a ‘best bet’ or non-negotiable aspect of schooling to be so seriously downgraded? More importantly, how can we learn from these mistakes? One could contend that we have ‘thrown the baby out with the bathwater’ and are now training a new profession to manage many of the ills of society, including those stemming from low levels of physical activity during the growing years. Much more needs to be done to educate the population regarding the profession. Any straw poll of community members would indicate that most have no idea of the scope of practice, role, and distinctive contribution of those involved in Exercise and Sport Science. Unfortunately, the same may be true regarding public knowledge and understanding of many health professions. Great opportunities may be realised if we offered high-quality Health and Physical Education programs across the school years and employed an inter- and multidisciplinary mindset to our work. [7]

My professional career has included strong involvement with a range of organisations, including the International Council of Sport Science and Physical Education (ICSSPE), the International Federation of Sports Medicine (FIMS), and Sports Medicine Australia (SMA). I also had a longstanding commitment to the once prominent but now discontinued, International Council for Physical Activity and Fitness Research (ICPAFR). Significant scientific contributions and friendships stemmed from scientific meetings of ICPAFR, including with Franco Viviani, Albrecht Claessens, Oded Bar-Or, Toivo Jürimäe, Willem van Mechelen, Neil Armstrong and many others. Important contributions were also made to the International Conference on Diet and Activity Methods (ICDAM), previously the International Conference on Dietary Assessment Methods. This included the brokering of a new focus on both diet and physical activity methodology commenced at ICDAM6 (Copenhagen, 2006) (Berit Heitmann, Lauren Lissner, and Anna Winkvist), a fine example of interdisciplinary success subsequently reflected in ICDAM7 (Washington DC, 2009) (Amy Subar), ICDAM8 (Rome, 2012) chaired by Barbara Burlingame at FAO, and ICDAM9 (Brisbane, 2015) chaired by me. However, the predominant focus of my work has been with the obesity fraternity, nationally and internationally.

Over the years, I served in a range of executive roles before being elected as the first non-medical President of the Australasian Society for the Study of Obesity (ASSO), the forerunner of the Australian and New Zealand Obesity Society (ANZOS). In this capacity, I was heavily involved in preparations for the 10^th^ International Congress on Obesity (ICO Sydney, 2006) and the preceding Satellite Conference on Physical Activity and Obesity (Brisbane, 2006), along with close collaborator and life partner, Nuala Byrne. Together, we have explored numerous research questions, most related to nutritional physiology and adaptation to weight loss in people with obesity. A primary goal during this work has been to remember the sage advice of Marcel Proust, that “the real voyage of discovery consists not in seeking new landscapes but in having new eyes”. Nuala’s passion for advancing knowledge in the field by questioning traditional maxims is exemplary, and from numerous examples, includes exploration of the shortcomings of the metabolic equivalent (MET) and acknowledgement that one size does not fit all. [8]

Over many years, books and book chapters dominated my scholarly outputs, a reflection of my somewhat non-traditional career. I wrote relatively few papers until my mid-30s with academic life focused on teaching and service. Early books included the first edited text on *Exercise and Obesity* (Smith-Gordon, first published in 1994) [9] with Mark Wahlqvist and contributions from leading international scientists. I am also particularly proud of other early work with Czech colleague, Jana Pařízková, including two editions of the comprehensive *Childhood Obesity: Prevention and Treatment* (CRC Press, 2001; 2005). [10, 11] Later, with Nuala and Neil King, we edited *Physical Activity and Obesity* (Smith-Gordon, 2006) [12] in conjunction with the ICO2006 Satellite Conference, and the following year with ICSSPE, *Children, Obesity and Exercise* (Routledge, 2007). [13]

If I was to shortlist the European cities with the greatest impact across my career, the standouts would surely be Prague and Vienna. I’ve been fortunate to spend significant blocks of time in both places, writing tracts of the *Childhood Obesity* books in Prague, and working with colleagues in the Nutrition and Health-Related Environmental Studies Section at the International Atomic Energy Agency (IAEA). Opportunities to serve the international community through research projects and Expert Missions related to obesity, body composition and energy expenditure assessment, particularly on behalf of the IAEA, can be traced to an invitation to present at the WHO Expert Meeting on Childhood Obesity (Kobe, Japan, 2005) and subsequent collaboration with Najat Mokhtar, now Deputy Director General and Head, Department of Nuclear Sciences and Applications, IAEA. The same meeting forged other important research collaborations, including with Anoop Misra and colleagues (then All India Institute of Medical Sciences [AIIMS] and now, Fortis Centre for Diabetes, Obesity and Cholesterol [C-DOC]), and a long history of work in South Asia. I have thoroughly enjoyed and much appreciated being ‘accepted’ by other esteemed colleagues in the region including Jeya Henry, Mario Soares, the late Prakash Shetty, Anura Kurpad, Ismail Noor and PhD scholars and Postdoctoral Fellows who have achieved great success in their own careers, including Ranil Jayawardena, Chathuranga Ranasinghe, and Masaharu Kagawa. [14]

My collaboration with Jana Pařízková has been particularly impactful, including recognition of her body of work, encompassing two editions of *Nutrition, Physical Activity and Health in Early Life* (CRC Press, 2010; 2017). [15, 16] Weaving threads from the past and acknowledging the contributions of others remains a poignant theme, highlighted again for me recently in reading the powerful accounts in *The Golden Maze: A Biography of Prague* (2020) [17] by Richard Fidler. To move forward, we need to move full circle to replicate important features of earlier generations. Too many early contributions in the growth and development, nutrition and physical activity fields have been forgotten, sometimes because of not being as readily accessible in today’s digital world. Acknowledging maxims from the past was also a focus of one of our earlier papers. [18]

In 2001, I joined Jan Borms and Marcel Hebbelinck (Free University of Brussels) and later, Tim Noakes (University of Cape Town), as a Series Editor of the prestigious Medicine and Sport Science Series (S. Karger AG, Basel). Since then, we have co-edited a long list of volumes after personally completing two earlier prominent titles with Jana Pařízková (Vol. 43, *Physical Fitness and Nutrition During Growth*, 1998) [19] and Toivo Jürimäe (Vol. 44, *Body Composition Assessment in Children and Adolescents*, 2001). [20] In 2006–2007, I had the pleasure of joining an international team of experts who delivered the IAEA Nobel Peace Prize Fund Nutrition Schools in Kampala, Uganda and Dhaka, Bangladesh on the double burden of malnutrition. I’ve also had the good fortune of serving on many other IAEA-funded regional projects and trained numerous Research Fellows from developing countries on the use of stable isotopes in nutrition with the support of the IAEA and the Australian Nuclear Science and Technology Organisation (ANSTO). These included a six-nation Regional Technical Cooperation project on *Childhood Obesity Prevention in Asia* and a twelve-nation Collaborative Research Project on *Body Fat and Metabolic Risk in Pre-adolescents and Adolescents*. More recently, I have been a Principal Investigator and Australian site lead on the large international Multi-Centre Infant Body Composition Reference Study (MIBCRS) to develop global references for infant body composition to sit alongside the WHO Child Growth Standards.

## Reflections of the way life used to be!

Imagine for a moment if our respective communities aspired to be the healthiest in our country and by doing this, created a positive legacy for subsequent generations. Why wait for the problem of obesity to worsen? In the words of David Rothkopf, “sometimes a problem reaches a point of acuity where there are just two choices left: bold action or permanent crisis”. There is no better time than the present to sow the seeds to realise this goal by working more closely with young people to improve their health and wellbeing. Here, the words of the former President of the United States of America, Theodore Roosevelt resonate, “what we do for ourselves dies with us. What we do for our communities lives long after we are gone”.

This reminds me of the relevance of the words, ‘reflections of … the way life used to be!’ from the classic hit song Reflections by the Supremes (1966). Our focus must be to build on existing capacity of our communities and in so doing, provide the foundation for the next generation to flourish and be healthier than their parents and grandparents. This aspiration is essential if we are serious about challenging ballooning childhood obesity statistics and the dire forecast by Olshansky et al. [21] Without “effective population-level interventions to reduce obesity … the youth of today may, on average, live less healthy and possibly even shorter lives than their parents”. Is this how we want to be remembered for the way we nurtured, (or not), our greatest assets, the next generation? In the words of Nelson Mandela, “there can be no keener revelation of a society’s soul than the way in which it treats its children”.

Young children are the key to the future with nutrition and physical activity being so critical that we must do everything possible not to restrict their movement. Health and education are central to the future of our children but sadly, too many are robbed of their developmental potential. Physical activity can be described as an underestimated investment in human capital with numerous developmental markers linked to appropriate levels of activity. [22] Physical literacy, the repertoire of movement skills necessary for an individual’s meaningful engagement in physical activity, and consistent with the establishment of fundamental movement (or motor) skills (FMS), is one of the most important life skills. However, as too many young children have a poor start to life characterised by unhealthy activity and eating behaviours, novel approaches are urgently needed to establish optimal conditions for the development and progression of FMS, and sound nutritional practices.

Imagine tackling the problem head-on globally within early education and care settings using a targeted and multi-pronged approach including collaboration between educators, teachers, families, and children. The realisation of baseline FMS would be a major step towards all children flourishing and reaching their physical health and education potential. Even better if across the school years, foundational FMS was capped with a high-quality Health and Physical Education curriculum, as referenced earlier.

Many parents happily accept the premise that children would benefit from a greater proportion of the school curriculum being devoted to academic pursuits (commonly at the expense of Health and Physical Education and the arts). Much less attention has been paid to the demise of physical activity across the curriculum and the resultant impact on health and wellbeing. Trudeau and Shephard [23] concluded that with competent delivery, physical activity can be added to the school curriculum by taking time from other subjects without compromising academic achievement. Adding time to ‘academic’ subjects by taking time from physical education does not guarantee an increase in grades—however the reverse is true, it is very likely to be detrimental to a young person’s health and wellbeing.

A focus on prevention is essential along with translation of current evidence or ‘best bets’ into practice; ideally utilising multi-level, whole-of-government, and multi-sectoral approaches. Sustainable changes are needed across all sectors including education, health, and transport, along with rigorous program evaluation. Everyone has a role to play, it is everyone’s responsibility.

The first 1000 days from conception to 2 years of age, or even better, the first 2000 days to 5 years of age, hold the key to the prevention of unhealthy weight gain. Indeed, I contend that infancy and the early childhood years constitute a critical and under-utilised life stage for obesity prevention. For maximum impact during these years of growth and development, physical activity and nutrition must return to being a higher priority along with physical environments conducive to active play. [24]

The minimum investment should see consistent education and support of parents and teachers, and the propagation of community nutrition and activity opportunities, including walking to and from school. When I was at primary school, albeit it many moons ago, everyone walked or cycled to and from school, and often back and forth from home for lunch. Why can’t the same be true today, at least for a significant proportion of young children? Concerns regarding safety from increased vehicular traffic and ‘stranger danger’ are the most common excuses used to limit engagement in such an important physical activity opportunity. The flipside is that if everyone walked or cycled to and from school, safety in numbers would be assured, particularly if oversight was provided by rostered adults, including parents and/or grandparents.

In the 1990s, we were lauded as progressive and innovative for the design, promotion, and delivery of Walk to School programs in Queensland primary schools, a simple and daily form of physical activity I took for granted during my formative years. A sad indictment of present-day society is that we needed a sophisticated training program for walking leaders and comprehensive insurance coverage for the Walk to School program to operate!

**Fig. 1:**
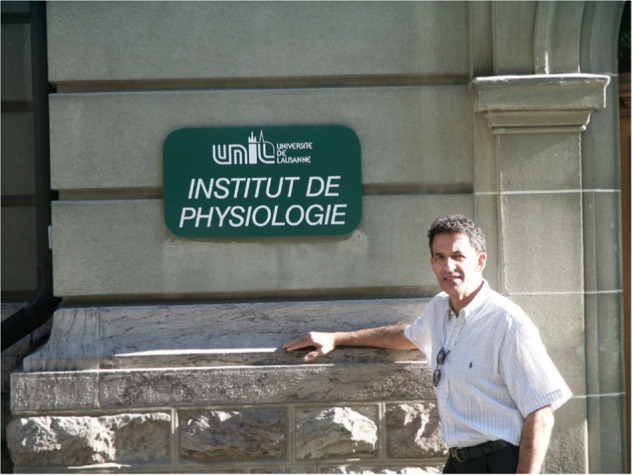
University of Lausanne, Switzerland. The author: Lausanne, Switzerland (circa 2007).

One of the best international active transport initiatives I have experienced is the Swiss walking school Pedibus (https://pedibus.ch/fr/) seen firsthand on regular sojourns to the Institute of Physiology, University of Lausanne to work with Yves Schutz and Dominique Durrer (see Fig. 1). In many parts of Switzerland, it is commonplace for approximately 75% of children from 4 years of age to walk to school ‘on’ the Pedibus supervised by parents and/or senior volunteers. What a wonderful intergenerational example of physical activity. There is absolutely no reason such an approach could not be replicated in many places across the globe.

In my opinion, one of the more counterproductive features of obesity research has been a preoccupation by some to determine whether nutrition or physical activity is the major driver of the obesity pandemic. As physical activity and eating behaviours ‘cluster’, we should address both together in obesity prevention efforts. Parents, siblings, and the family environment, along with early childhood education and care settings and schools, are all critically important. A new baseline may include encouragement and support of ‘small changes’ in diet and/or physical activity as modest changes are more likely to be tolerated by the greater proportion of individuals. [25]

Other ‘best bets’ for obesity prevention should encompass greater opportunities for young mothers and families to gain the knowledge and skills necessary to maximise health and wellbeing. Such knowledge and understanding would help adults and caregivers be better placed to support the healthy growth and development of their offspring. Consistent with increasing evidence of the importance of preconception health to improved outcomes for the next generation, strategies to optimise the health and wellbeing of adolescents would be a great investment in the future. [26, 27] I am particularly honoured to be a Chief Investigator on the Centre for Research Excellence Health in Preconception and Pregnancy (CRE-HiPP) working with Helen Skouteris and colleagues (https://hipp.org.au/). Other initiatives I am passionate about in the space include the wonderful work undertaken by colleagues as part of the B4 Early Years Coalition (https://b4.education.tas.gov.au/). Given the very strong evidence that quality early health and education has such an integral bearing on long-term outcomes, imagine if the first 2000 days of life was everyone’s responsibility!

To move forward with impact and make a dent in the number of children living with obesity globally, we would be wise to acknowledge some of our earlier failings. This might include reconceptualising our intervention approaches, recognising the collective assets of our communities, and systematically focusing on the co-creation of shared value. Such approaches have been variously described, including as asset-based community development (ABCD) and more recently, collective impact, where a strengths (as opposed to deficit-based) approach, is employed. A strengths-based approach encompasses the co-design or co-creation of workable, relevant, place-based initiatives with increased likelihood of sustainability, particularly in more resource-poor settings.

In North-West (NW) Tasmania, we have been working to improve the health and wellbeing of residents in three sentinel site communities using such a capacity building approach. The region has often been described as one of the most picturesque and visually appealing locations in the state, and one of the primary food bowls. Rich and productive volcanic soils guarantee fine vegetables and fruit and support high-quality dairy herds and Wagyu beef cattle. Sadly, people in this region are often maligned for their poor health outcomes and low educational attainment, a situation diametrically opposed to the idyllic physical environment.

Interestingly, the NW Coast also has a rich and proud history of sport and engagement in habitual physical activity, but the health profile of the present-day population is characterised by high levels of overweight and obesity at all ages. How is it that such dichotomies exist? How could we have got things so wrong, and more importantly, how could we possibly make a difference now? The overarching goal of our project, CAPITOL (Critical Age Periods Impacting the Trajectory of Obesogenic Lifestyles) (https://www.utas.edu.au/health/community-programs/capitol), is to work with community to co-design place-based approaches to intervention. Such an undertaking is uncommon compared to the mainstream practice of introducing favoured or road-tested interventions parachuted into communities to ‘solve’ challenges. In our experience, there is often a lack of appropriate co-designed, collaborative, and community-led relevant information. Priority initiatives include approaches to supporting parents and educators across the first 1000 days, including nutrition and activity, and school gardens and nutrition embedded in the curriculum being the norm rather than exception.

The notion of ‘free range’ children resonates with me as this was certainly the nature of the childhood I experienced, replete with opportunities to be physically active. We have transitioned from this to a generation of ‘backseat’ kids, commonly transported everywhere, including to and from school. Many parents are not happy unless they can drop their child as close to the front door of the school as possible. It appears that the preference for some would be to drive into the school grounds, up the front steps, to drop their child off at the classroom door! It should be little surprise that the greatest daily accident risk for children coincides with school drop-off and pick-up times.

Michael Young, the influential social innovator, who developed a multitude of social enterprises, including the Open University, had some poignant advice regarding schools and schooling. Young described traditional education, including higher education, as a ‘diet thought up by educators about what they (children) should have.’ According to Young, being ready to learn was a much more important factor than assuming all children would learn in the same way and on the same timetable. This thinking also reminds me of the Finnish education system and is exemplified in the writing of Pasi Sahlberg. Along with Michael Doyle, Sahlberg wrote the informative book, *Let the Children Play: How more Play will Save our Schools and help Children Thrive*, [28] and illustrates how we have lost sight of what is important for many children. My strong suggestion is that active play should be featured in the school curriculum, even better if positive play experiences co-existed with opportunities for children to grow, harvest, and cook with bounty nurtured in school and community gardens.
